# Reversible Neutrophilia Driven by Monoclonal Gammopathy of Clinical Significance Simulating Chronic Neutrophilic Leukemia: A Case Report

**DOI:** 10.1155/crh/7405586

**Published:** 2026-08-20

**Authors:** Éliott Larin, Lambert Busque, Genevieve Chabot-Roy, Sylvie Lesage, Gaspard Jadot, Richard LeBlanc, Jean Roy, Jean-Sébastien Claveau, Natasha Szuber

**Affiliations:** ^1^ Faculty of Medicine, University Laval, Québec City, Canada; ^2^ Department of Hematology, Maisonneuve-Rosemont Hospital, Montreal, Canada, maisonneuve-rosemont.org; ^3^ Faculty of Medicine, University of Montreal, Montreal, Canada, umontreal.ca; ^4^ University Institute of Hematology-Oncology and Cell Therapy, Maisonneuve-Rosemont Hospital, University of Montreal, Montreal, Canada, maisonneuve-rosemont.org; ^5^ Maisonneuve-Rosemont Hospital Research Centre, Montreal, Canada, maisonneuve-rosemont.org; ^6^ Département de Microbiologie, Infectiologie et Immunologie, Université de Montréal, Montreal, Canada, umontreal.ca

**Keywords:** case report, chronic neutrophilic leukemia, granulocyte colony–stimulating factor, monoclonal gammopathy of clinical significance, neutrophilia

## Abstract

We encountered a 51‐year‐old male with a family history of acute myeloid leukemia who presented with persistent neutrophilia and splenomegaly progressing over eight years. Despite extensive workup, no myeloid driver mutation was identified and repeated bone marrow biopsies revealed no clonal myeloid disorder. However, an IgA kappa plasma cell clone was detected, consistent with monoclonal gammopathy of clinical significance (MGCS). Reactive neutrophilia secondary to the abnormal plasma cell clone was suspected, hypothesized to be mediated by paraneoplastic granulocyte colony–stimulating factor (G‐CSF) secretion. G‐CSF levels were elevated before treatment, supporting the hypothesis that it might induce neutrophilia. The patient received antimyeloma therapy and exhibited significant hematologic improvement: reduction in neutrophil count, normalization of G‐CSF levels, and improvement in splenomegaly. This is the first report to describe improvement of neutrophilia following antimyeloma therapy, providing novel evidence that MGCS could simulate chronic neutrophilic leukemia (CNL).

## 1. Introduction

Neutrophilia is a common phenomenon, frequently caused by secondary etiologies, such as infection or inflammatory processes. In contrast, clonal neutrophilia, such as CNL, is rare. CNL is a *BCR::ABL1*‐negative myeloproliferative neoplasm (MPN), characterized by persistent neutrophilia, splenomegaly, and the presence of *CSF3R* mutations in most cases [1].

Monoclonal gammopathy of undetermined significance (MGUS) is a plasma cell disorder defined by the presence of a serum monoclonal protein < 30 g/L and clonal bone marrow plasma cells < 10%, in the absence of organ damage [2]. Although typically asymptomatic, MGUS carries an annual risk of progression of approximately 0.5%–1% to multiple myeloma (MM) or related malignancies [2]. In some cases, the term MGCS is used to describe a monoclonal protein–producing clone that, while not meeting criteria for malignancy, causes clinically relevant organ damage or systemic complications [3].

Although both neutrophilia and MGUS are common entities in hematology, their co‐occurrence, particularly when neutrophilia is not attributable to a secondary cause, is uncommon [4]. Cases of concurrent MM and CNL have been reported [5, 6], with affected patients generally exhibiting a poorer prognosis compared to either disease occurring alone, necessitating a distinct therapeutic approach [5]. Rarely, MM has been shown to induce secondary, nonclonal neutrophilia through paraneoplastic production of G‐CSF. This phenomenon is uncommon and may confound the diagnosis by mimicking a concomitant myeloid neoplasm, such as CNL [5].

We report herein a rare case of a patient with chronic neutrophilia evolving over 8 years. Although initially suspected to represent a clonal MPN, the patient was ultimately found to have an underlying MGCS driving excessive neutrophil production, via paraneoplastic G‐CSF secretion. To the best of our knowledge, the association of MGUS/MGCS with reactive neutrophilia is exceedingly rare. Given the limited number of published reports and the lack of clinical guidelines, this case provides valuable insight into the diagnostic approach and therapeutic management of such a clinical presentation.

## 2. Case Presentation

**FIGURE 1:**
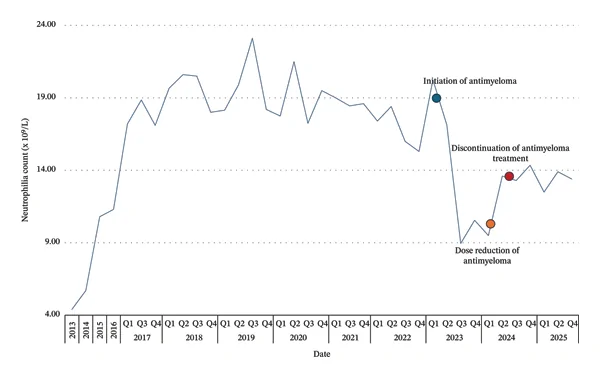
Neutrophil counts over time. Neutrophil counts are shown as mean values per quarter. The timeline indicates the periods before, during, and after treatment.

A 51‐year‐old Caucasian male with a medical history of gout and a family history of acute myeloid leukemia presented to his primary physician in 2017 with neutrophilia that had been progressing over the past 2 years. His white blood cell count (WBC) was 13.8 × 10^9^/L in 2015 and 21.7 × 10^9^/L in October 2017, while the neutrophil count rose from 5.7 × 10^9^/L in 2014 to 19.3 × 10^9^/L in 2017 (Figure 1). Peripheral blood smear revealed toxic granulations and Döhle bodies. Initially referred to an infectious disease specialist for evaluation of a possible underlying infectious etiology, all investigations including blood cultures, viral serologies, and parasitic screens were negative. A PET scan revealed diffuse bone marrow hypermetabolism and mild, homogeneous splenomegaly (14 cm) without evidence of active infection or neoplasia. The patient was subsequently referred to a hematologist in December 2017.

At that time, the patient denied fever, chills, early satiety, or weight loss reporting only occasional night sweats (fewer than five episodes over the past year). Physical examination was unremarkable. The differential diagnosis included CNL, myelofibrosis, myeloproliferative neoplasm‐unclassifiable (MPN‐U), and reactive neutrophilia. A bone marrow exam revealed hypercellular (95%) with granulocytic hyperplasia, 1% blasts, no evidence of myelofibrosis, and 1%–5% of plasma cells. Molecular testing was negative for *BCR::ABL1, JAK2, CALR, MPL,* and *CSF3R* mutations, and a 42‐myeloid gene panel using next‐generation sequencing showed no pathogenic variants. Cytogenetics revealed a normal male karyotype. Leukocyte adhesion molecule and endogenous colony formation assays were normal. Autoimmune screening, including antinuclear antibodies, rheumatoid factor, and anticyclic citrullinated peptide antibodies, was also all negative. Serum protein electrophoresis and immunofixation identified a nonquantifiable IgA kappa monoclonal protein, an involved kappa serum free light chain of 80.4 mg/L, and an uninvolved lambda free light chain of 11.5 mg/L with a ratio of 7.0. Total IgA level was within the normal range. Beta‐2‐microglobulin was slightly elevated at 3.04 mg/L. Hemoglobin and renal function were normal, while a whole‐body bone scan showed no lytic bone lesions. A diagnosis of MGCS IgA kappa was thus confirmed.

Despite persistent neutrophilia (18.4 × 10^9^/L in 2020), progressive thrombocytopenia (72 × 10^9^/L in 2020), and evolving splenomegaly (15 cm in 2020), no clonal marker could be detected to support a diagnosis of MPN or any other myeloid malignancy. Three repeat bone marrow exams (2020, 2021, and 2022) consistently showed granulocytic hyperplasia, interstitial infiltration by 2%‐3% monotypic IgA kappa plasma cells, and focal grade 1 myelofibrosis. Over the following years, what was initially a nonquantifiable IgA monoclonal spike evolved to a quantitative IgA (3.54 g/L) and kappa serum free light chains (340 mg/L, with a ratio of 39.7 in October 2022) increased steadily, accompanied by progressive splenomegaly (18.6 cm in November 2021), suggesting a potential plasma cell–driven process. At that time, inflammatory markers (C‐reactive protein and ferritin) remained within the normal range.

In November 2022, reactive neutrophilia secondary to the abnormal plasma cell clone was suspected, hypothesized to be mediated by paraneoplastic G‐CSF secretion. In March 2023, G‐CSF level was quantified at 105 pg/mL. The differential diagnosis for G‐CSF elevation includes reactive (infectious and inflammatory) causes, exogenous administration, and both hematologic and solid malignancies via paraneoplastic production and/or cytokine dysregulation. In this case, infectious and inflammatory sources were explicitly excluded through extensive diagnostic workup; the patient was not on exogenous therapy; and clonal myeloid/lymphoid as well as solid neoplasms were excluded through detailed hematologic workup (morphological/pathological, molecular biology, cytogenetics, flow cytometry, and so on) as well as comprehensive imaging.

To confirm the diagnosis and prevent complications, a therapeutic trial of an antimyeloma therapy was initiated on March 31st, 2023. The patient received four cycles of cyclophosphamide (300 mg/m^2^ weekly), bortezomib (1.5 mg/m^2^ weekly), and dexamethasone (Drd) (40 mg weekly) (CyBorD regimen) from March to June 2023. At the time of treatment initiation, bone marrow plasma cell infiltration remained limited to 2%‐3%, and the patient did not fulfill SLiM‐CRAB criteria for MM.

After the fourth cycle of CyBorD, response was consistent with stable disease and minimal clinical improvement. There was a mild improvement in platelet count (78 × 10^9^/L). However, the kappa serum free light chains remained largely unchanged at 287 mg/L, with an IgA level at 2.82 g/L. Splenomegaly progressed (20.1 cm in September 2023) and leukocytosis persisted (27.2 × 10^9^/L). Given this limited response, the treatment regimen was subsequently modified to daratumumab, lenalidomide, and DRd.

By December 2023, following 5 cycles of DRd, the patient exhibited significant hematologic improvement: IgA level decreased to 0.3 g/L, kappa serum free light chain decreased to 69.5 mg/L, and remarkably, WBC and neutrophil count decreased to 13.5 × 10^9^/L (from 21.9 × 10^9^/L pretreatment) and to 10 × 10^9^/L (from 19.3 × 10^9^/L pretreatment), respectively, while platelets increased to 129 × 10^9^/L (73 × 10^9^/L pretreatment). Splenomegaly also decreased (18.4 cm in February 2024) and signs of hypersplenism improved on clinical examination. These findings support the hypothesis that the plasma cell clone was driving the phenomenon of neutrophilia/splenomegaly, mimicking a concomitant MPN.

**FIGURE 2:**
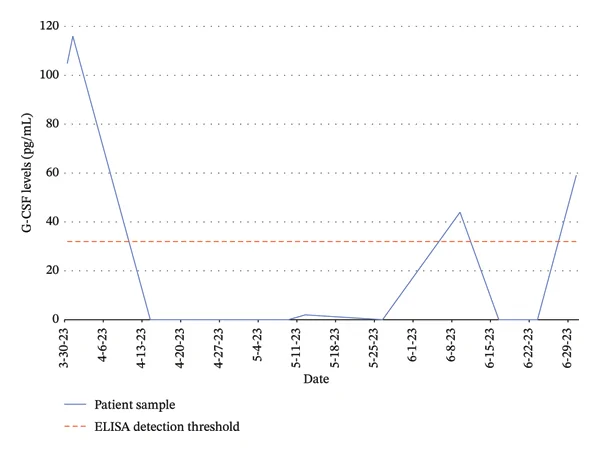
Serum G‐CSF levels over time.

During treatment, the patient reported fatigue, reduced appetite, and dysgeusia leading to DRd dose reduction in February 2024 and eventual discontinuation in May 2024. In October 2024, all side effects had resolved. Blood tests in June 2025 revealed a mild increase in neutrophils and monoclonal proteins, which currently have stabilized: neutrophil count was 13.4 × 10^9^/L, IgA level was 1.05 g/L, while kappa serum free light chain decreased to 41.3 mg/L. Abdominal ultrasonography in December 2024 showed stable splenomegaly (18.5 cm). Following treatment initiation, G‐CSF concentrations normalized rapidly and remained within physiological range thereafter, paralleling the patient’s clinical improvement (Figure 2).

## 3. Discussion

We report a rare case of secondary and nonclonal neutrophilia associated with splenomegaly driven by a MGCS through postulated plasma cell paraneoplastic production of G‐CSF. While our patient initially presented a phenotype suggestive of CNL, the absence of an activating *CSF3R* or other myeloid gene mutation and absence of associated bone marrow findings invalidate this diagnosis, as well as that of other MPN or myeloid malignancies. This case highlights the diagnostic complexity of chronic neutrophilia in the absence of MPN markers and underscores the importance of considering paraneoplastic cytokine production as a potential cause.

Importantly, elevated G‐CSF levels prior to treatment and their subsequent decrease following MGCS‐directed therapy supporting the role of G‐CSF as driver of a “CNL‐like” phenotype. Our patient achieved a very good partial response of his plasma cell dyscrasia with antimyeloma treatment, accompanied by concurrent improvement in neutrophilia, thrombocytopenia, and splenomegaly. While extensive testing supported the absence of alternative inflammatory or malignant drivers and delineated a strict temporal relationship between disease activity and G‐CSF levels, analyses localizing G‐CSF expression specifically within bone marrow plasma cells were not feasible in this case; thus, alternative cellular sources of G‐CSF cannot be definitively excluded, though they are considered highly unlikely. Additionally, FISH analysis for common plasma cell cytogenetic abnormalities was not performed given the low plasma cell burden.

Although very rare, most reported cases of neutrophilia secondary to monoclonal gammopathies have been observed in patients with MM [5]. In these reports, G‐CSF had been implicated in driving neutrophilia, which resolved following initiation of daratumumab‐based regimen [7, 8]. Our case further extends these findings by contextualizing this phenomenon for MGCS. While Nagai et al. [9] previously documented G‐CSF elevation in plasma cell neoplasm, our case illustrates this association with clone‐specific therapy (DRd) and formalizes this presentation within the MGCS framework. To our knowledge, only two other cases have described MGCS and reactive neutrophilia, hypothesized to result from paraneoplastic G‐CSF production [10, 11]. However, in neither case were serum G‐CSF levels measured, nor was the neutrophilia shown to be reversible with antimyeloma treatment. Our observations provide novel evidence that MGUS/MGCS could promote paraneoplastic, G‐CSF‐mediated neutrophilia, and suggest that antimyeloma regimens could effectively reverse this phenomenon.

## 4. Conclusion

Our case highlights several important clinical considerations. First, the presence of MGUS or MGCS should not be dismissed as incidental in patients with unexplained neutrophilia. Although rare, these plasma cell disorders should be considered in the differential diagnosis of unexplained neutrophilia in patients lacking typical myeloid mutations (e.g., *CSF3R*). Furthermore, measurement of serum G‐CSF may provide critical evidence to support the diagnosis and should be considered in such patients to corroborate involvement of this biological pathway. Recognition of this possible paraneoplastic association may allow clinicians to tailor therapy appropriately and avoid extensive workups for other causes of neutrophilia. Finally, therapeutic trials of antimyeloma agents may serve both diagnostic and therapeutic purposes in these settings.

## Author Contributions

Lambert Busque, Natasha Szuber, and Jean‐Sébastien Claveau conceived of and designed the study. Genevieve Chabot‐Roy and Sylvie Lesage performed laboratory analysis. Lambert Busque, Éliott Larin, Natasha Szuber, and Jean‐Sébastien Claveau were involved in data analysis, interpretation, and wrote the original draft which was then reviewed and approved by all co‐authors. All authors had access to the raw data. Lambert Busque and Jean Roy verified the data.

## Funding

No funding was received for this manuscript.

## Consent

The patient provided informed consent to participate in the Quebec MPN registry.

## Conflicts of Interest

The authors declare no conflicts of interest.
